# Towards effective outbreak detection: a qualitative study to identify factors affecting nurses’ early warning surveillance practice in Solomon Islands

**DOI:** 10.1186/s12913-018-3508-9

**Published:** 2018-09-10

**Authors:** Adam T. Craig, Cynthia A. Joshua, Alison R. Sio, Michael Lauri, John Kaldor, Alexander E. Rosewell, Gill Schierhout

**Affiliations:** 1 The Kirby Institute, University of New South Wales, Wallace Wurth Building, High St, Kensington, NSW 2052 Australia; 2 Solomon Islands Ministry of Health and Medical Services, PO Box 349, Honiara, Solomon Islands

**Keywords:** Disease outbreaks, epidemiology, public health surveillance, syndromic surveillance, surveys and questionnaires, knowledge attitude practice survey, communicable diseases, emerging, Pacific Islands, Solomon Islands, countries, developing

## Abstract

**Background:**

Intelligence generated by a surveillance system is dependent on the quality of data that are collected. We investigated the knowledge, attitudes and practices of nurses responsible for outbreak early warning surveillance data collection in Solomon Islands to identify factors that influence their ability to perform surveillance-related tasks with rigour.

**Methods:**

We interviewed 12 purposively selected surveillance nurses and conducted inductive analysis on resulting data.

**Results:**

Interviewees were knowledgeable and willing to contribute to the surveillance system. Constraining factors included the perception that surveillance was less important than patient care and could be ‘deferred’ during busy periods and wide variability in the application of case definitions. Motivating factors were frequent in-clinic training, formal recognition for good performance, incentives and designation of a focal point. Nurses held mixed views about the effect of mobile technologies on surveillance practice.

**Conclusions:**

This study identified several challenges to consistent and accurate data collection and reporting. Engagement of different parts of the health system, including human resources and health facilities’ management, is needed to address these challenges.

**Electronic supplementary material:**

The online version of this article (10.1186/s12913-018-3508-9) contains supplementary material, which is available to authorized users.

## Background

Disease surveillance is an essential function of public health, providing the mechanism to monitor population health and guide policy and program responses [1]. Inaccurate measures of disease through poorly performing surveillance systems may lead to sub-optimal decision making, wasted resources and poorer health outcomes [2–4].

The collection and analysis of routine clinical reports of syndromes in people presenting to health facilities, commonly known as ‘syndromic surveillance’, is a simple and cost-effective strategy used by many countries to detect communicable disease outbreaks [5–8]. The validity of intelligence generated by syndromic surveillance depends, in part, on the quality of the primary data collected, which is influenced by the knowledge, attitudes and practices of those responsible for data collection, typically front-line nurses [1, 3, 9].

With a per-capita gross national income of USD 1561 and a United Nations Human Development Index ranking of 156 of 188 nations [10], Solomon Islands is considered one of the least developed countries in the world [11–13]. Communicable diseases remain significant contributors to the health burden in Solomon Islands, causing an estimated 8% of all deaths [14] and more than 10% of disability-adjusted life years lost in 2015 [15]. Since 2011, Solomon Islands has implemented a syndromic surveillance system modelled on the broader the Pacific Syndromic Surveillance System (PSSS) [6, 16]. The Solomon Islands Syndromic Surveillance System (SI-SSS) collects weekly count data for five syndromes (acute fever and rash, influenza-like illness, dengue-like illness, acute diarrhoea and prologued fever) in patients presenting to ten sentinel health facilities. At each facility, surveillance is managed by a senior nurse. This nurse acts as the SI-SSS focal point for the facility and is responsible for overseeing the surveillance practices of between three and five nurses on roster each shift. The paper-based system depends on nurses to accurately apply case definitions and report data centrally.

While other researchers have explored knowledge, attitudes and practices about surveillance among doctors [17, 18], surveillance coordinators [17, 19], infection control officers [20] and community members [21] there is scant understanding about the knowledge and views of nurses with respect to their early warning surveillance roles and the impact these have on their practice. With communicable outbreak-prone diseases significant contributors to the burden of disease in most developing countries and nurse-based syndromic surveillance a much relied upon early detection strategy, this study aims to fill a knowledge gap by identifing factors that support and undermine surveillance practice and hence the quality, and ultimately utility, of data on which outbreak intelligence is based.

## Methods

### Data collection tool

Drawing on previous surveillance-related knowledge-attitude-practice (KAP) studies [9, 21–23] we developed a semi-structured questionnaire. We designed the tool to elicit information about respondents’ (i) demographic profile; (ii) access to resources to perform surveillance tasks; (iii) knowledge of the SI-SSS’s purpose and process; (iv) attitude, motivations and barriers in relation to syndromic surveillance; and (v) syndromic surveillance-related data collection and reporting practices (see Additional file 1). We pilot-tested the questionnaire with staff of two health centres in Honiara (Solomon Islands capital) to determine its acceptability, comprehensibility, and time required to implement. Questions probing perceptions of electronic data collection were added in response to a theme emerging from the pilot phase. A convenience sample was used to select the pilot sites. Data collected from pilot sites were included in the analysis. Health facility data (such as facility catchments and out-patient throughputs) were obtained from the Solomon Islands Ministry of Health and Medical Services (MHMS) Health Information Office and population data was sourced from the National Statistics Office [24].

### Study sites and sample selection

While effort was made to collect data from all ten sentinel site that contribute to the SI-SSS for various logistical reasons we were only able to collect data from seven. These sites (termed ‘study sites’) included the National Referral Hospital (NRH) (375 beds, ~ 1654 out-patients/week), three rural hospitals (92 beds, ~ 410 out-patients/week; 137 beds, ~ 636 out-patients/week; 33 beds, ~ 505 out-patients/week, respectively), and three Honiara-based community clinics (0 beds, ~ 391, ~ 568 and ~ 610 out-patients/week, respectively). Within each study site, respondents were purposively selected so a range of surveillance roles were represented in the sample. Respondents were required to have participated in SI-SSS for at least 6-months.

The three other sentinel sites that contribute to the SI-SSS but not involved in the study were all rural hospitals with 43, 40 and 20 in-patient beds and approximately 170, 200 and 160 out-patients throughput/week, respectively.

### Data analysis and management

Interview data were collected on an electronic form designed in GoSurvey [25] and audio recorded for transcription. Using a general inductive approach [26], we designed a coding frame that categorised data as related to ‘knowledge’, ‘attitude’ and/or early warning surveillance ‘practice’, and as an ‘enabling’ or ‘constraining’ factor. Verbatim quotes highlighting emergent themes were extracted from the transcriptions. Descriptive analyses including calculation of proportions, standard deviations and inter-quartile ranges was calculated using Microsoft® Excel 2016.

## Results

A total of 12 respondents (four general nurses, six general nurses with facility management responsibilities; and two infection control nurses) were interviewed (Table 1). Ten (83%) respondents were interviewed in person and two (17%) by telephone.

**Table 1:** Demographic profile and results of the knowledge-attitude-practice survey of nurses responsible for early warning surveillance data collection and reporting

	Community clinics		National Referral Hospital		Rural hospitals		Total	
	No.	Percent	No.	Percent	No.	Percent	No.	Percent
Number of respondents	7	58%	2	17%	3	25%	12	100%
Sex								
Female	6	50%	2	17%	1	8%	9	75%
Male	1	8%	0	0%	2	17%	3	25%
Role								
Nurses	4	33%	1	8%	0	0%	5	42%
Nurse/facility managers	3	25%	1	8%	1	8%	5	42%
Nurse/ surveillance focal point	0	0%	0	0%	2	17%	2	17%
Qualification								
Tertiary nursing qualifications	7	58%	2	17%	3	25%	12	100%
Years involvement with SI-SSS^a^								
< 1 year	1	8%	0	0%	0	0%	1	8%
1–4 years	2	17%	0	0%	3	25%	5	42%
> 4 years	4	33%	2	17%	0	0%	6	50%
Knowledge of functions of disease surveillance^b^								
Able to describe at least one function	3	43%	0	0%	3	100%	6	50%
Able to describe > 1 function	4	57%	2	100%	0	0%	6	50%
Knowledge of key objective of SI-SSS	7	100%	2	100%	3	100%	12	100%
Willingness to contribute to the SI-SSS								
Very willing	7	100%	2	100%	3	100%	12	100%
Access to the internet from personally owned devise								
Yes	4	57%	1	50%	1	33%	6	50%
No	3	33%	1	50%	2	67%	6	50%
Self-reported familiarity with using the internet								
High level	3	43%	2	100%	3	100%	8	67%
Moderate level	3	43%	0	0%	0	0%	3	25%
None/limited level	1	14%	0	0%	0	0%	1	8%
Leading factors that motivate nurses to conduct surveillance	In-clinic visits from MHMS staff to provide semi-formal and opportunistic trainings (*n* = 8; 67%) Formal recognition by supervisors and senior staff / awards for performance (*n* = 6; 50%) Seeing ones’ data presented in the weekly surveillance reports (*n* = 6; 50%)							
Leading barriers that inhibit robust surveillance practice	Lack of time when clinic case load is high (*n* = 12; 100%) Perception that surveillance is not nurses’ responsibility but rather conducted voluntarily (*n* = 10; 83%) Surveillance seen as a secondary / less important task than clinical work (*n* = 10; 83%) Extended delays between facility visits / in-service trainings (*n* = 4; 33%) Perception that data that is provided is not being used/used efficiently (*n* = 2; 17%)							

^a^Solomon Islands Syndromic Surveillance System; ^b^ As stated in [27]

Respondents had been involved in the SI-SSS for a median of 4.5 years (IQR: 2.8–5.3 years) with three (25%) involved since the system’s inception. The majority (75%) of respondents were female. All had tertiary nursing qualifications.

The collective population catchment of the study sites was estimated at 34.4% of Solomon Islands’ population, and the mean weekly outpatient throughput of sites was 419 patients (SD: 191 patients).

### Access to resources for surveillance

Resources aiding participation in surveillance include access to surveillance case definitions and data collection forms, and a means to transmit data to health authorities [27]. Respondents from the NRH and two of the rural hospitals reported access to multiple telephone lines, a computer with internet connectivity and printers and faxes. Two respondents (one from NRH and one from a rural hospital) had access to the internet through a personal device (Table 1). The respondent from the other rural hospital reported that her facility had a telephone (that was restricted to receive incoming calls only) and no computer or internet access. This respondent had a tablet computer with internet connection capability, however, cost, poor network coverage and connection speed precluded internet access from this device. All community clinics had a landline telephone (although one clinic reported the line did not work at all times). None had a computer or internet access although four (57%) staff (including at least one staff from each clinic) had internet access from a personal device, such as a mobile phone.

The community clinics relied on the MHMS surveillance unit for consumable resources (e.g., reporting forms) while the hospitals could produce their own. All forms were standardised.

### Knowledge of surveillance purpose and process

Knowledge of the purpose of surveillance was generally high with all respondents having attended at least one syndromic surveillance-focused training and able to describe at least one of the WHO identified functions of surveillance [28], six (50%) could describe more than one function, and none could describe all four. Respondents from rural sites had a slightly greater knowledge of the functions than those from urban sites. All respondents knew that the primary purpose of the SI-SSS is the early detection of outbreaks; eight (67%) could identify other purposes including monitoring disease trends over time and location (*n* = 6; 50%), monitoring the progress of outbreaks once identified (*n* = 3; 25%), and to inform hospital surge planning (*n* = 2; 17%) (Table 1).

### Attitude, motivations and barriers to surveillance

Attitudes towards participation in syndromic surveillance were overwhelmingly positive with all respondents reporting a high level of willingness to contribute to the system. Despite this positive attitude, 10 (83%) reported that they viewed surveillance as a ‘secondary’ task undertaken in good faith and that it was not as important as their patient care role (Table 1). A typical response was, “when it gets busy surveillance are [sic] one of the first things to be drop”.

Respondents from rural sites expressed a strong commitment to collect and deliver surveillance data and resolve to overcome obstacles; “here we are committed to contribute [to the SI-SSS]. Even when I am away, or the power is down or something, we always find a way to get our data to them [the MHMS surveillance unit]”.

When asked, what motivates nurses to participate in the SI-SSS, the most commonly identified factor was face-to-face engagement and training provided by the national surveillance coordinator during scheduled and opportunistic in-clinic visits (*n* = 8 respondents; 67%). This was followed by designation of a local person to support and monitor surveillance practices within a facility (*n* = 7 respondents; 58%); formal acknowledgement for work done (*n* = 6 respondents; 50%); seeing ones’ data presented in the weekly surveillance reports (*n* = 6 respondents; 50%); financial incentives (not currently offered) (*n* = 5 respondents; 42%); and non-monetary rewards, such as opportunities to attend trainings (*n* = 2 respondents; 17%) (Table 1). The value accorded to face-to-face engagement and training was explained by a view that these engagements demonstrated management’s commitment to the SI-SSS and respect for nurses’ contribution to it.

While some (42%) suggested the introduction of financial incentives would be a motivator, others felt additional pay was unwarranted and that surveillance tasks should be considered a core function of a nurse’s job. When explored further it became clear that some externally and well-funded public health programs (such as the Global Fund-supported tuberculosis and malaria programs) employed staff at a higher remuneration to undertake similar surveillance tasks while other programs (including the SI-SSS) did not. This situation appears to have created mild animosity among some and a sense that additional financial incentives to collect data for the SI-SSS was warranted. This sentinent was illustrated by an interviewee who said, “in the beginning when asked to do [syndromic] surveillance we saw others [i.e., staff of other public health programs] getting laptops and training and more paid when it wasn’t being offered to us”. They added, “we felt that we should be compensated if asked to do additional work that fills up our day”. Another respondent said “we sometimes delay or forget to do surveillance as we are paid as nurses, not as surveillance officers”. The issue of compensation may have influenced the extent to which some nurses prioritised surveillance tasks.

Respondents commented that when motivation fell, so too did their commitment to collecting data. Motivation to perform surveillance tasks was reported to wane when facilities’ caseload was high (*n* = 12 respondents; 100%), when periods between MHMS clinic visits were lengthy (*n* = 4 respondents; 33%) and when there was a perception that the data being collected were not used efficiently (*n* = 2 respondents; 17%) (Table 1).

### Surveillance practices

Different approaches to applying case definitions were identified across the study sites. Respondents from hospital sites reported determining whether signs and symptoms in presenting patients met a surveillance case definition by retrospective (at the end of the reporting week) review of free text entries in the ‘diagnosis’ and ‘treatment’ fields in facility-kept treatment logbooks. Typically, the free text descriptions were 1–5 words and insufficient to independently ascertain whether the case presentation met a surveillance definition. When questioned how ambiguity was addressed, respondents described personal interpretation; cross-referencing different data fields in clinic logbooks (e.g., reports of “diarrhoea” that mentioned treatment of “fluid replacement” was assumed by one respondent to meet the acute diarrhea case definition of “>3 loose stools within 24 hours”); and through checking back with the nurse that made a logbook entry as strategies applied to collect more information.

Surveillance practice at the three community clinics in Honiara was slightly different with MHMS staff physically visiting each facility to undertake the logbook review and case extraction process. While recognised as an approach intending to lessen the workload of facility staff, some nurses felt undermined as a result. One respondent said, “I don’t know why they [the MHMS] come here to collect the data. It is like they don’t trust us to be able to do the job”.

Weekly tallied data were transferred to the MHMS by phone (from sites outside of Honiara); by hand delivery (from NHR); or collected during site visits (from community clinics). Email delivery was uncommon owing to difficulties accessing a computer, slow internet speeds (i.e., download/download speeds of ~ 0.04/~ 819.2 Mbps) and cost. Facsimile or short message service (SMS) messaging were not used. Some respondents expressed interest in integrating mobile technologies as part of the SI-SSS, others were less enthusiastic citing personal capacity, logistical and cost concerns.

## Discussion

This study identifies constraints and enablers to nurses’ ability to perform data collection and reporting tasks required for outbreak early warning syndromic surveillance.

Interviewing staff from seven of the ten facilities that contribute the SI-SSS allowed us to explore KAPs across sites. While we found strong concordance among interviewees about the motivating and limiting factors that influence surveillance practice we found slightly higher levels of knowledge and enthusiasm for surveillance amongst rural respondents. This may be due to a greater self-reliance among rural workers, and a greater sense of responsibility towards their surveillance roles.

### Formalising knowledge and understanding for better data collection

In broad terms, we found that appreciation of the purpose of early warning surveillance was high among nurses. This is supported by findings of past evaluations in the Pacific context [22, 23]. However, we found a general under-appreciation for the rationale for rigorous application of case definitions and a commonly held perception that surveillance activities were of secondary importance to clinical roles, and to be deprioritised when workloads increased.

The application of retrospective case identification methods at all sites points to the need for a deeper understanding by those collecting data of the need for consistency and rigour to ensure data quality, and hence system’s integrity, is maintained. The finding that in-facility visits from MHMS system coordinators were highly valued suggests that interventions to strengthen data collection should occur at all sites. Periodic provision of support to nurses responsible for surveillance, along with monitoring of data quality and feedback about sites’ performance is needed.

Our findings that nurses perceived that surveillance tasks were being performed in good faith, and the role financial incentives may play in motivating surveillance practice suggests that these issues require attention from MHMS leadership and a clear policy direction.

### Addressing procedural barriers

Ideally, early warning surveillance data capture would occur at the time of patient consultation when attending nurses are best placed to apply surveillance case definitions correctly. Application at this time opens the possibility for more frequent data reporting and analysis, and hence more timely outbreak detection and response. The finding that case identification relied on the retrospective application of case definitions suggests that data collection processes need to be reviewed. Limited ability to influence how clinic records are kept may preclude the streamlining of surveillance data collection practices within health facilities and impede ‘best practice’ procedures in this and similar settings. Further research is needed to determine likely public health impacts of the current data capture processes, and to inform the development of standard methods for case identification and recording, if retrospective data capture is to be retained.

Advances in Solomon Islands’ health information systems more broadly may provide opportunities to modify surveillance procedures and to better integrate these with routine data collection at the facility level, thus reducing duplication of data captured in outpatient settings.

### Maintaining motivation

The finding that surveillance was seen as of secondary importance to nurses’ clinical roles and was sometimes neglected suggests the need to implement strategies to maintain the motivation of nurses with regard to their surveillance tasks. Motivating factors identified in this study suggest that greater acknowledgement of nurses’ contribution to the SI-SSS, and clear communication about how data are being used are warranted. Strategies to ensure nurses receive adequate recognition for their contribution is relatively straightforward and inexpensive and would build goodwill and motivation. Potential actions include explicit public acknowledgements; awards for good practice; provision of ‘soft’ incentives, such as opportunities to attend training; and the inclusion of site-specific data in weekly analysis.

### Use of technology to improve surveillance

Although we did not assess the validity of data collected, the inconsistent and delayed data collection processes identified in this study suggests data quality may be compromised. The finding that nurses had mixed views about the adoption of mobile technologies as part of surveillance practice, coupled with the uneven access to and familiarity with internet-enabled devices suggests a cautious approach will be needed if technology were to be introduced.

Mobile technology has been successfully used as part of early warning surveillance in several developing settings [27–30]. Owing to the similarities Solomon Islands shares with neighbouring Papua New Guinea (PNG), attention should be paid to the work of Rosewell et al. [30] who report PNG’s experience using mobile devices and Global Information Systems for malaria surveillance. Emerging evidence from PNG and other comparable settings should be explored.

Taking a more health systems perspective, it is important that introduction of new technology to enhance the SI-SSS builds on, seamlessly integrates with and strengthens broader efforts in Solomon Islands to improve health information management, including interoperability with the newly introduced electronic health data management platform, District Health Information System version-2 [29]. In countries like Solomon Islands, electronic health information systems, robust mobile data capture from peripheral sites, and household and village-level mapping applications could be integrated with DHIS2 for enhanced outbreak surveillance.

### Limitations of the study

This study has several limitations. First, the small number of respondents, while indicative of the workforce, somewhat limits the generalisability of the findings. Secondly, while we interviewed staff from 70% of sites we were not able to cover all, and respondents were weighted towards senior nurses from urban settings who were perhaps not able to reflect the views of junior or rural-based staff. Finally, approximately half of the interviews were conducted in group settings where interpersonal dynamics may have influenced participants’ candour. Despite these limitations, the strength of this study lies in its exploratory nature, providing insight into factors that enable and inhibit nurses’ data collection practice not previously identified.

## Conclusion

Our study found that while general knowledge about the purpose and practice of surveillance was high and attitudes towards participating in the SI-SSS were positive among nurses numerous deeper knowledge, perception, and practical factors pose challenges to consistent and accurate data collection. Attitudes towards the use of mobile technology for data reporting were varied and while potentially beneficial a cautious approach to its introduction is required. Practical actions to addressing challenges identified in this paper should be framed within the Solomon Islands Government’s broader agenda to strengthen the country’s health information system.

## Additional file

Additional file 1: Knowledge-attitude-practice study interview data collection form. (PDF 113 kb)

## Abbreviations

DHIS2: District Health Information System, version 2
KAP: Knowledge, attitude and practice
Mbps: Megabytes per second
MHMS: Ministry of Health and Medical Services
NRH: National Referral Hospital
PNG: Papua New Guinea
SI-SSS: Solomon Islands Syndromic Surveillance System
SMS: Short messaging service
SSS: Syndromic surveillance system
USD: United States Dollars
