# Supplementary material for: Towards effective outbreak detection: a qualitative study to identify factors affecting nurses’ early warning surveillance practice in Solomon Islands
Source: BMC Health Serv Res. 2018 Sep 10;18:702. doi: 10.1186/s12913-018-3508-9 (PMC6131946; doi:10.1186/s12913-018-3508-9)
Supplement: Supplementary file 1 — Knowledge-attitude-practice study interview data collection form. (PDF 113 kb) [file 12913_2018_3508_MOESM1_ESM.pdf]

KNOWLEDGE-ATTITUDE-PRACTICE STUDY INTERVIEW DATA COLLECTION FORM

**ADMINISTRATION**		
1	Date	[Date format]
2	Location	[Options list]
3	Interviewer	[Options list]
4	Consent form number	[Number]
5	Permission to record	O Yes<br>O No
**DEMOGRAPHICS**		
6	Initials (do not identify interviewee)	[Free text]
7	Sex	O Male<br>O Female
8	Role	O Nurse<br>O Nurse manager<br>O Facility manger<br>O Other…
9	Highest education	O College / university<br>O High school<br>O Other…
10	Years of experience with SI-SSS	[Number]
**CLINIC INFRASTRUCTURE FOR SURVEILLANCCE**		
11	Does the facility have a functioning phone (landline or cell phone) at all times?	O Yes, all the time<br>O Yes, but not all the time<br>O No<br>O Don't know
12	Does the facility have a functioning computer?	O Yes<br>O No<br>O Don't know
13	Is there access to internet within the facility today?	O Yes<br>O No<br>O Don't know
14	Do you have experience using the internet?	O Yes, a lot<br>O Yes, a little<br>O No
15	Do you have private access to the internet?	O Yes, on phone / tablet<br>O Yes, on computer<br>O No
16	How many days it the clinic open?	[Number]
17	How many days does the clinic offer general out-patient clinic?	[Number]
18	On average, how many patients are seen by the clinic?	[Number]
19	How many clinical staff that have a role in surveillance working at the clinic?	[Number]
**KNOWLEDGE OF SURVEILLANCE PURPOSE AND PRACTICE**		
20	What do you see as the main functions of a disease surveillance system?	[Free text]
21	What do you understand to be the main purpose of the SI-SSS?	[Free text]
22	Do you see the SI-SSS having any other (secondary) purposes?	[Free text]
22	How accurate to you feel the current burden of infectious diseases in your area is captured by the SI-SSS?	O 1 Not at all<br>O 2<br>O 3<br>O 4<br>O 5 Very accurate
	Why do you say this? How could it be improved?	[Free text]
23	How useful do you feel the SI-SSS is for identify outbreaks?	O 1 Not at all<br>O 2<br>O 3

		O 4<br>O 5 Very useful
**ATTITUDE AND MOTIVATION**		
24	In general, how do you feel about the SI-SSS?	[Free text]
25	Personally, how do you feel about being asked to collect data for the system?	[Free text]
26	What, if anything, makes your surveillance role challenging or difficult?	[Free text]
27	What do you see has / would motivate nurses to participate in the SI-SSS?	[Free text]
29	Do you have any other suggestions for ways staff may be encouraged / supported to collect data for the SI-SSS?	[Free text]
30	What discourages staff from participating in the SI-SSS?	[Free text]
31	Overall, how well do you think SI EWSS is performing regarding is primary purpose of detection outbreaks?	O 1 Poorly<br>O 2<br>O 3<br>O 4<br>O 5 Very well
**DATA COLLECTION AND REPORTING PRACTICE**		
32	Do you have written copies of the SSS case definitions?	O Yes, on display in clinic<br>O Yes, but not on display<br>O No
33	In your view, are the case definitions clear (unambiguous)?	O Yes<br>O No
34	Do you (or the facilities staff) have any problem applying the case definitions?	O Yes<br>O No
35	If yes, what are they?	[Free text]
36	In your view, how consistently are the case definitions applied by staff at your facility?	O 1 Poorly<br>O 2<br>O 3<br>O 4<br>O 5 Very well
	If not consistently, what inhibits their use?	[Free tex]
37	At what point (time) are case definition applied?	O When patient is seen<br>O At the end of the day<br>O At the end of the week by clinic staff<br>O At the end of the week by MHMS<br>O Don't know<br>O Other….
38	If at the end of the day/week, how are case definition applied accurately?	[Free text]

39	In your view, how accurate is the count of case reported by your facility?	O 1 Very poor<br>O 2<br>O 3<br>O 4<br>O 5 Very accurate
40	Is data ever checked for its accuracy?	O Yes<br>O No<br>O Don't know
41	How is surveillance data transmitted to MHMS?	O Phone call<br>O Email<br>O SMS/TXT<br>O Delivered in person<br>O Picked up by MHMS
42	How reliable is this method?	O Very<br>O Somewhat<br>O Not very / poor
43	What inhibits (gets in the road) of on time data collection and reporting?	[Free text]
**ELECTRONIC DATA COLLECTION**		
44	Do you see value in electronic data transfer of EWSS data?	O Yes, a lot<br>O Yes, some<br>O No
45	If yes, what value do you see?	[Free text]
46	What barriers do you see?	[Free text]
47	Would it be feasible to report data more frequently?	O Yes, daily<br>O Yes, twice weekly<br>O No
48	If not, why?	[Free text]
49	Do you have any other comments you'd like to add?	[Free text]
**FEEDBACK**		
50	Would you like to receive feedback on the results of this study?	O Yes<br>O No
51	If Yes, provide contact details	
